# Co-Occurrence and Habitat Use of Fin Whales, Striped Dolphins and Atlantic Bluefin Tuna in the Northwestern Mediterranean Sea

**DOI:** 10.1371/journal.pone.0139218

**Published:** 2015-10-12

**Authors:** Robert Klaus Bauer, Jean-Marc Fromentin, Hervé Demarcq, Blandine Brisset, Sylvain Bonhommeau

**Affiliations:** 1 IFREMER (Institut Français de Recherche pour l’Exploitation de la MER), Resarch Unit Marbec (Marine Biodiversity, Exploitation and Conservation), Sète, France; 2 IRD (Institut de Recherche pour le Développement), Resarch Unit Marbec (Marine Biodiversity, Exploitation and Conservation), Sète, France; Hellenic Centre for Marine Research, GREECE

## Abstract

Different dolphin and tuna species have frequently been reported to aggregate in areas of high frontal activity, sometimes developing close multi-species associations to increase feeding success. Aerial surveys are a common tool to monitor the density and abundance of marine mammals, and have recently become a focus in the search for methods to provide fisheries-independent abundance indicators for tuna stock assessment. In this study, we present first density estimates corrected for availability bias of fin whales (*Balaenoptera physalus*) and striped dolphins (*Stenella coeruleoalba*) from the Golf of Lions (GoL), compared with uncorrected estimates of Atlantic bluefin tuna (ABFT; *Thunnus thynnus*) densities from 8 years of line transect aerial surveys. The raw sighting data were further used to analyze patterns of spatial co-occurrence and density of these three top marine predators in this important feeding ground in the Northwestern Mediterranean Sea. These patterns were investigated regarding known species-specific feeding preferences and environmental characteristics (i. e. mesoscale activity) of the survey zone. ABFT was by far the most abundant species during the surveys in terms of schools and individuals, followed by striped dolphins and fin whales. However, when accounted for availability bias, schools of dolphins and fin whales were of equal density. Direct interactions of the species appeared to be the exception, but results indicate that densities, presence and core sighting locations of striped dolphins and ABFT were correlated. Core sighting areas of these species were located close to an area of high mesoscale activity (oceanic fronts and eddies). Fin whales did not show such a correlation. The results further highlight the feasibility to coordinate research efforts to explore the behaviour and abundance of the investigated species, as demanded by the Marine Strategy Framework Directive (MSFD).

## Introduction

Despite having different habitat preferences, marine top predators often co-occur. In fact, different cetacean and tuna species are well known for their associative behaviour [1]. For example, yellowfin tuna (*Thunnus albacares*) have been described to associate with several dolphin species, a behaviour that has been utilized by tuna purse seiners in the eastern tropical Pacific to locate tunas [2]. While such associations are assumed to reduce predation risks, and may provide foraging benefits, they are apparently temporary and driven by the environment [3]. Tuna-dolphin associations in the eastern tropical Pacific appear to be linked to a compression of the tuna’s vertical habitat, induced by a shallow thermocline in this region, which covers a thick hypoxic oxygen minimum zone [3]. In other areas, such as the Azores, feeding aggregations of tunas, dolphins and sea birds are often observed around shallow seamounts during summer, representing sites of enhanced biological productivity [4; 5]. Other environmental conditions, including persistent oceanographic fronts are also known to structure the marine environment and aggregate marine predators [6; 7]. Associations of surface feeding short-beaked common dolphins (*Delphinus delphis*) and ABFT have been also reported from the Maltese Islands (35% of dolphin sightings) during May and July, probably linked to seasonal movements in relation to prey availability [8].

Improved knowledge on the factors influencing the co-occurrence of marine predators can help enhance our understanding of habitat use, which is crucial for conservation management [9]. Multi-species monitoring programs, identifying hot-spot areas, can provide important elements of this information. Aerial surveys represent a common tool to investigate the density and abundance of marine mammals. This method has also recently come under focus in the search for methods to provide fisheries-independent abundance indicators for tuna stock assessment [10]. In this context, an annual aerial survey program was initiated in 2000 to monitor juvenile ABFT abundance in the GoL in the Northwestern Mediterranean Sea [11]. This area represents an important nursery ground for ABFT. It is characterized by a large continental shelf, multiple canyons, large runoff from the Rhone river, and high frontal and eddy activity. It is located to the west of the Pelagos Sanctuary, a Marine Protected Area (MPA) that contains breeding and feeding habitats of several endangered cetacean species, of which striped dolphins and fin whales are the most abundant [12; 13]. In fact, both species are frequently observed during tuna aerial surveys. However, little is known of their abundance in the GoL and similarly, of the co-occurrence of ABFT with other marine predators, i.e. cetaceans. ABFT apparently associate less frequently with dolphins than yellowfin tuna, although mature ABFT sometimes participate in mixed-species feeding aggregations around the Azores [4]. By contrast, some reports indicate the use of cetacean sightings by purse seine fishermen to locate ABFT in the Mediterranean Sea [14; 15]. Furthermore, dolphins have occasionally been reported as bycatch in the ABFT purse seine fishery in the Mediterranean, although at very low numbers [16; 17; 18].

In this study, we investigate patterns of spatial co-occurrence and density of these three species in the GoL, based on aerial survey data, and analyze them in terms of known species feeding preferences and environmental characteristics (i. e. mesoscale activity) in the survey area.

## Materials and Methods

### Aerial surveys

**Fig 1:**
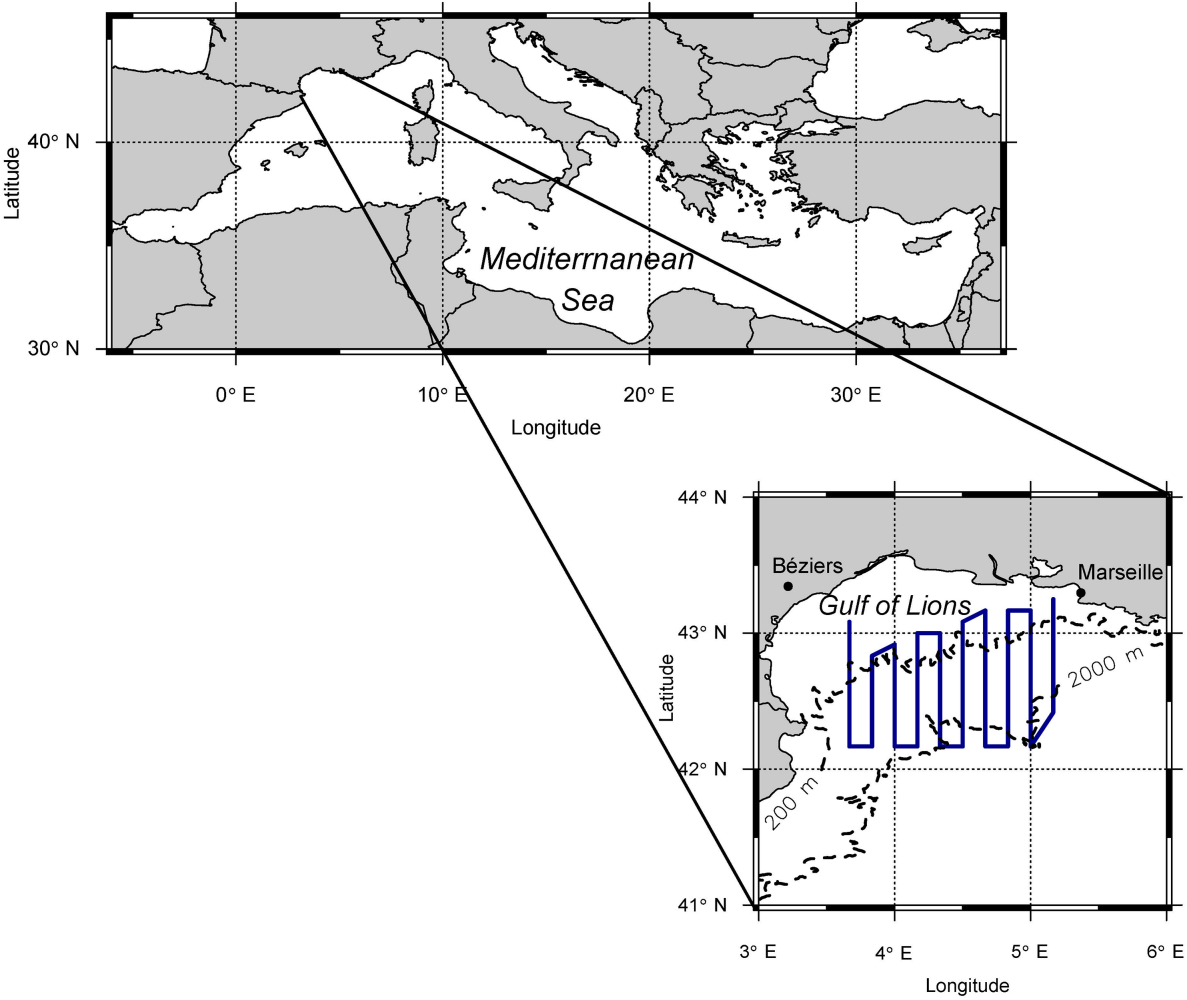
Study area and transect lines of aerial surveys (blue). The dashed lines represent the 200 and 2000 m isobaths, indicating the continental shelf break of the Gulf of Lions.

Aerial surveys targeting ABFT were carried out in the GoL from August to October between 2000 and 2003 and from 2009 until present [11]. During this season juvenile ABFT are particularly abundant in this area [19]. Apart from ABFT, a variety of cetacean species, notably fin whales and striped dolphins, and less frequently pilot whales (*Globicephala melas*), Rissos’s dolphins (*Grampus griseus*), short-beaked common dolphins and bottlenose dolphins (*Tursiops truncatus*), were sighted during the aerial surveys. Striped dolphins and the morphologically very similar short-beaked common dolphins could not always be distinguished, but as the latter has apparently almost vanished from the Northwestern Mediterranean Sea, we assume that such sightings exclusively represent striped dolphins [20; 21; 22]. A standard survey consisted of 10 survey transects, placed randomly in North-South direction over the study area, covering primarily the shelf break of the Gulf of Lions, which corresponds to the historical fishing ground for juvenile ABFT in this region. To achieve statistical validity, approximately 4 replicate aerial surveys were conducted during each survey year. Starting points and time breaks between replicates (usually 1–2 weeks) were selected based on weather conditions. A full randomization of starting points, tested in 2000, proved not to be feasible since the weather conditions, in particular the wind strength, were often quite different between the western and eastern part of the survey area (Tramontane and Mistral winds). Flights were conducted around noon to avoid sun glare and only under suitable weather conditions (< 28 km/h wind, no clouds or whitecaps). Surveys were performed with a Cessna C 337 “Push Pull” from 2000 to 2011, while a Cessna 208 ISR has been used since 2012. The change in airplanes was motivated by the greater range of the Cessna 208 ISR that allowed us to fly all 10 transects of a standard survey during one day without refueling (Fig 1; total length: 1120 km; off-effort inter-transect distance: 13.8 km). By contrast, the fuel capacity constraints of the first airplane required that the route be split into a western and an eastern component, which were surveyed independently, usually during subsequent days. Average speed was 200 km/h with an operational altitude of 300 and 500 m above sea level, for the first and second plane respectively. One to three trained observers were on board each flight. Both planes were not equipped with bubble windows, usually used to assure that no objects/animals are missed directly below the aircraft. In order to reduce this bias, pilots were instructed to focus on the transect line, thus providing additional sighting information. For each sighting event, its GPS position as well as the number of sighted animals (i.e. ABFT and cetacean schools) were recorded and documented photographically, if possible. The Cessna 208 ISR was further equipped by a WESCAM MX-15HDi camera with built-in GPS that allowed sighted objects to be georeferenced from the transect line. Conversely, during previous flights with the Cessna C 337, GPS positions of sighted animals were taken directly above the sighting locations. This required the plane to leave its route and, after recording school positions, to return to its former position on the transect line. Perpendicular distances of animal locations to the transect line were then calculated based on additionally recorded flight tracks, disregarding off-route excursions. Another, more common sampling practice in aerial surveys, where the positions of sighted objects are not directly measured, is to back-calculate perpendicular distances to sighted objects from sighting angles and the aircraft altitude [23; 24]. Here, sighting angles are measured by an inclinometer while the object of interest is abeam the aircraft. As this method requires additional handling time by the observer, it was not applicable for our aerial surveys, conducted at a high traveling speed, during which time cetacean and tuna schools can pass through the detection range in a few seconds, often in swift succession.

In addition to the position, number and size of tuna schools, the observers on board the aircraft and the weather conditions (e.g. clouds, sea state) were recorded for each survey. Transect sections with heavy cloud cover or breaking waves were skipped and therefore discarded from subsequent analysis. For more details on the survey design see [11].

### Estimating cetacean and juvenile bluefin tuna densities

#### Detectability and line transect modeling

Striped dolphin and fin whale densities in the GoL region were estimated using the line transect approach [25]. Using this method a detection function that describes the decline in the detection probability with increasing distance was fitted to the sighting frequencies per perpendicular distance. Knowing the probability of detection (detectability), the percentage of missed objects can be calculated, allowing the total object/animal abundance to be extrapolated over a relatively large area, assuming that all objects are available for detection (see subsection on “Availability and perception bias”). Furthermore, potential effects on the detectability due to other variables can be assessed in the line transect approach, increasing the reliability of abundance and density estimates [26]. In this study, the combination (team) and number of observers on board, the number of individuals per sighting event (school size), the sea state in the area (Beaufort scale) and the plane used, were considered as potential covariates in the line transect modeling. For example, dolphin schools are probably more likely to be detected with increasing school size and more observers on board. By contrast, unfavourable weather conditions, even though avoided, could significantly impede the detectability (e.g. by whitecaps). The selection of the covariate “plane” in the modeling of the detectability of dolphin schools was considered following the change in the operational altitude due to the change in airplane in 2012.

After careful inspection of the sighting frequencies, observations were right-truncated in order to facilitate the modeling of detectability [25], discarding 5% and 10% of the largest distances (≧ 2.45 km) of dolphin and (≧ 2.56 km) fin whale sighting locations, respectively. Secondary sightings, made during off-route excursions, can affect abundance and density estimates since they are related to additional (secondary) survey effort. Such events were not systematically recorded during our aerial surveys, and hence could not be totally excluded in this analysis. However, they were very rare, and generally consisted of only small schools of cetaceans or tunas detected during large off-route excursions. Related effects were therefore assumed to be minor and further reduced by truncating distant sightings. Not using bubble windows can reduce visibility beneath the aircraft and thus lead to a lack of detections in the area close to the transect line. Such effects impair the modeling of the detectability and sighting frequencies are often left-truncated [25]. However, our data did not indicate a lack of detections close to the transect line for any of the species analyzed. We therefore refrained from such an operation.

The modeling of the detection function was conducted using the multi covariate distance sampling (MCDS; [27; 28]) engine of the *“ddf”*-function of the R-package *“mrds”* [29]. Models were selected based on the Akaike’s information criterion (AIC) and further evaluated using goodness of fit tests (q-q plots, Cramer-von Mises and Kolmogorov-Smirnov, and Chi-square tests; [30]). The density of striped dolphin and fin whale schools were then calculated per survey year, with

$$\begin{array}{c} {\hat{D}}_{a}=\frac{n_{i}}{2wLP_{a}}, \end{array}$$ (1)

giving the number of *n* sightings per km^2^ in the combined area of both sides of a transect with a length *L* and within truncation width *w*, thereby accounting for changes in the detectability *P*_*a*_. To facilitate comparison with other studies, we also calculated the sighting rate (SR), which is defined as

$$\begin{array}{c} {\hat{SR}}_{i}=\frac{n_{i}}{L}, \end{array}$$ (2)

the numbers of sightings per km transect. The SR does therefore not account for changes in the detection probability *P*_*a*_ (like the strip transect approach). It further neglects the constant 2*w* in the denominator, as it is usually applied when sighting distances are unknown, thus potentially incorporating sightings over large distances.

Sighting rates and densities of cetacean schools were calculated based on the pooled datasets per survey year, using the “*dht*”-function of the R-package *“mrds”* [29]. Absolute dolphin and fin whale densities (Ind/km^2^), as well as corresponding sighting rates (Ind/km) were also calculated, multiplying former school estimates (Eqs 1 and 2) with the average school size per survey year. Coefficients of variation (CV) and 95% log-normal confidence intervals (CI) of density estimates were obtained using a Satterthwaite-approximation for degrees of freedom as implemented in the “*dht*”-function [25; 29]. The calculated cetacean density estimates were then compared with those of juvenile ABFT from the same aerial surveys [11] and literature values on the densities of the investigated cetacean species in the Mediterranean Sea.

#### Availability and perception bias

Line transect modeling relies on the assumption that all animals are detected on the transect line (at distance zero; 25). However, in the field this assumption may not completely apply. Marine animals are not always visible to observers (e.g. due to submergence; availability bias), and even if so, they can be missed by the observers on the transect line (perception bias) due to various factors (e.g. school size, swell, traveling speed, observation angle as well as observer platform, experience or fatigue). The availability bias refers to the combined probability that the animals of interest are located at the surface or are “surfacing” while the observer passes. It hence depends on the surface behaviour of animals and the traveling speed of the observer. This probability can be estimated by

$$\begin{array}{c} a\left(S,x\right)=\frac{E\left(sf\right)}{E\left(sf\right)+E\left(d\right)}+\frac{\hat{w}\left(x\right)-\hat{w}{\left(x\right)}^{2}E{\left(d\right)}^{-1}0.5}{E\left(sf\right)+E\left(d\right)}, \end{array}$$ (3)

where *E*(*sf*) refers to the average duration of surfacing and *E*(*d*) to the average duration of submergence [31]. The term $\hat{w}\left(x\right)$ represents the maximal duration during which an animal is in the observer’s view. As this “time window” for newly surfacing animals is increasing with perpendicular distance *x*, so does the availability probability. It is defined by

$$\begin{array}{c} \hat{w}(x)=\frac{x}{v}[(\cot\phi_{1}+\cot\phi_{2})], \end{array}$$ (4)

where *v* gives the aircraft speed, and *ϕ*_1_ and *ϕ*_2_ correspond to the lateral obstruction angles that limit the view of the observer to the front and rear [32]. The availability probability per perpendicular distance *a*(*S*, *x*) can then be used to derive corrected density estimates, by applying the corrected detection probabilities *P*_*c*_ in Eq 1:

$$\begin{array}{c} P_{c}(x)=P_{a}(x)a(S,x) \end{array}$$ (5)

These estimators have been applied to other aerial surveys in the western Mediterranean Sea, including surveys on striped dolphin abundance [32; 33]. For an aerial survey conducted along the nearby Spanish Mediterranean coastline, Gómez De Segura et al. [33] estimated an availability probability *a*(*S*, 0) of 0.676 (*SE* = 0.1632) for small schools (≦ 15 individuals) and 1 for larger schools of striped dolphins (always visible to observers). The underlying data on dive and surface intervals of striped dolphins was thereby obtained from an auxiliary boat survey. Considering the slightly higher speed at which our surveys were conducted, we recalculated the availability probability for striped dolphins. Besides the average duration of surface and dive intervals, we also applied the same lateral obstruction angles as in [32] (35° to the front and 40° to the rear). In fact, their aerial surveys were also performed with a Cessna C 337 “Push Pull” aircraft with no bubble windows. Viewing conditions in the Cessna 208 ISR were considered to be quite similar. To our knowledge, availability bias has not yet been evaluated for fin whales in the Mediterranean Sea. However, the duration of surface and dive intervals of fin whales were assessed during a boat survey, conducted during June—August in 1995–1997 in the Ligurian Sea [34], which is located to the northeast of our study region. In this study, surface and dive intervals lasted on average 90 s (±34.6 SD) and 227.5 s (±133.3 SD), respectively, for the period before the boat was approaching the whales. As no comparable data for the rather small fin whale schools (on average < 2 individuals) was available to us, we generally applied these values to estimate the availability probability of fin whales per perpendicular distance.

ABFT density estimates provided by Bauer et al. [11] are not corrected for availability bias. Such a correction is probably more complicated, as tunas are not obliged to regularly frequent the water surface, e.g. for breathing. In fact, the diving behaviour of ABFT and hence surface availability may change significantly depending on environmental conditions [35; 36; 37]. This question is part of current research, but remains to be answered.

Perception bias is usually assessed with double-observer platforms using mark-recapture distance sampling (MRDS) [38; 39]. Since no such platform was available to us during the aerial surveys, perception bias could not be accounted for, for any of the species studied.

Cetacean density estimates corrected for availability bias were compared with uncorrected estimates of the three species and literature estimates from cetacean studies in the western Mediterranean Sea.

### Spatial distribution and co-occurrence

In order to identify core areas of cetacean and ABFT occurrences in the GoL, we interpolated sighting locations per year on a square grid of 500x500 points (horizontal resolution: 6.7 km), using a fixed kernel density estimation algorithm (KDE; [40]). More precisely, we applied a bivariate normal kernel, given by the “kde2d” function from the R-package “MASS” [41] To facilitate interpolation between sighting locations, we selected a bandwidth (search radius) of 0.5 degrees, which is approximately four times the average inter-transect distance. In order to simplify comparison, kernel density estimates were rescaled to percentages. Based on annual estimates obtained in this manner, average spatial densities and their standard deviation during the survey period were calculated. In addition, the yearly overlap of core distribution areas (50% kernel density) of all three species was assessed. Furthermore, the co-occurrence of fin whales, striped dolphins and ABFT during the aerial surveys was assessed by calculating the percentage of sighting positions with co-occurring species within a radius of 2.5 km.

### Ocean fronts

Oceanic mesoscale structures, particularly ocean fronts often show increased levels of biological productivity and thus are known to attract top predators such as tunas [6; 7]. Royer et al. [6] demonstrated the affiliation of ABFT in the survey region with SST and chlorophyll-a (chla) fronts in the GoL based on sighting locations, made during the aerial surveys. Since we focus on the locations of core areas of cetaceans and ABFT in the GoL, we investigated average, and not daily, front locations during the survey period. This bears the advantage that persistent front locations are selected that are more likely to represent signals of biological productivity. To do so, we used SST and chla data with a spatial resolution of 4 km from the Moderate Resolution Imaging Spectroradiometer (MODIS-Aqua), available on the NASA ocean color web server (http://oceancolor.gsfc.nasa.gov/). We calculated front locations for all survey dates from 2002–2012 (MODIS-Aqua started transmitting in June 2002), using average satellite images from +/- 3 days, thus maximizing data coverage, which can otherwise be impaired by clouds. To do so, we applied the front detection algorithm presented by Nieto et al. [42]. Average front locations of both data sets, SST and chla, were subsequently compared with core sighting areas of all three species.

## Results

A total of 93 fin whales (70 sightings) were observed during the 8 survey years (Table 1). Sightings usually consisted single individuals (72.9%). Only occasionally two or three whales were observed at the same time, accounting 21.4 and 5.7% of all sightings made, respectively. Such sightings often included mothers with their calves. Fin whales were almost exclusively sighted on the continental shelf break. Only one individual was observed on the shelf area. Availability estimates indicated that only around 25% of fin whales were located at the surface during the aerial surveys and thus available for detection.

**Table 1:** Summary table of aerial surveys conducted in the GoL during 2000–2012 with the total length of effectuated transect route, the number of sighting events per species with number of on-effort sightings in parentheses.

Year	Effort (km)	Survey days (n)	Sighting events (n)		
			Fin whales	Striped dolphins	Blue fin tuna
2000	2876.1	6	2 (2)	14 (14)	49 (34)
2001	3933.5	8	11 (9)	-	59 (49)
2002	4425.0	9	11 (11)	22 (20)	48 (39)
2003	5512.1	11	8 (6)	28 (26)	100 (91)
2009	3692.3	8	6 (6)	20 (18)	178 (167)
2010	2417.2	6	10 (6)	25 (20)	86 (68)
2011	4304.7	9	13 (7)	43 (41)	235 (214)
2012	3709.4	5	9 (8)	28 (26)	267 (238)
2000–2012	30870.3	62	70 (60)	180 (165)	1022 (900)

During the survey period 14 to 43 dolphin schools were sighted each year. Dolphin sighting data from 2001 were incomplete and therefore excluded from this analysis. Dolphins were usually observed in schools of varying size (Fig A in S1 File). School size estimates ranged between 1 and 200 individuals, with an average of 30 individuals. Annual fluctuations in dolphin school size were very pronounced with relatively few large schools found in 2012 despite the similar survey effort. In comparison with fin whales, availability of striped dolphins was relatively high (> 70% at the surface), and thus related effects on density estimates were rather small.

**Fig 2:**
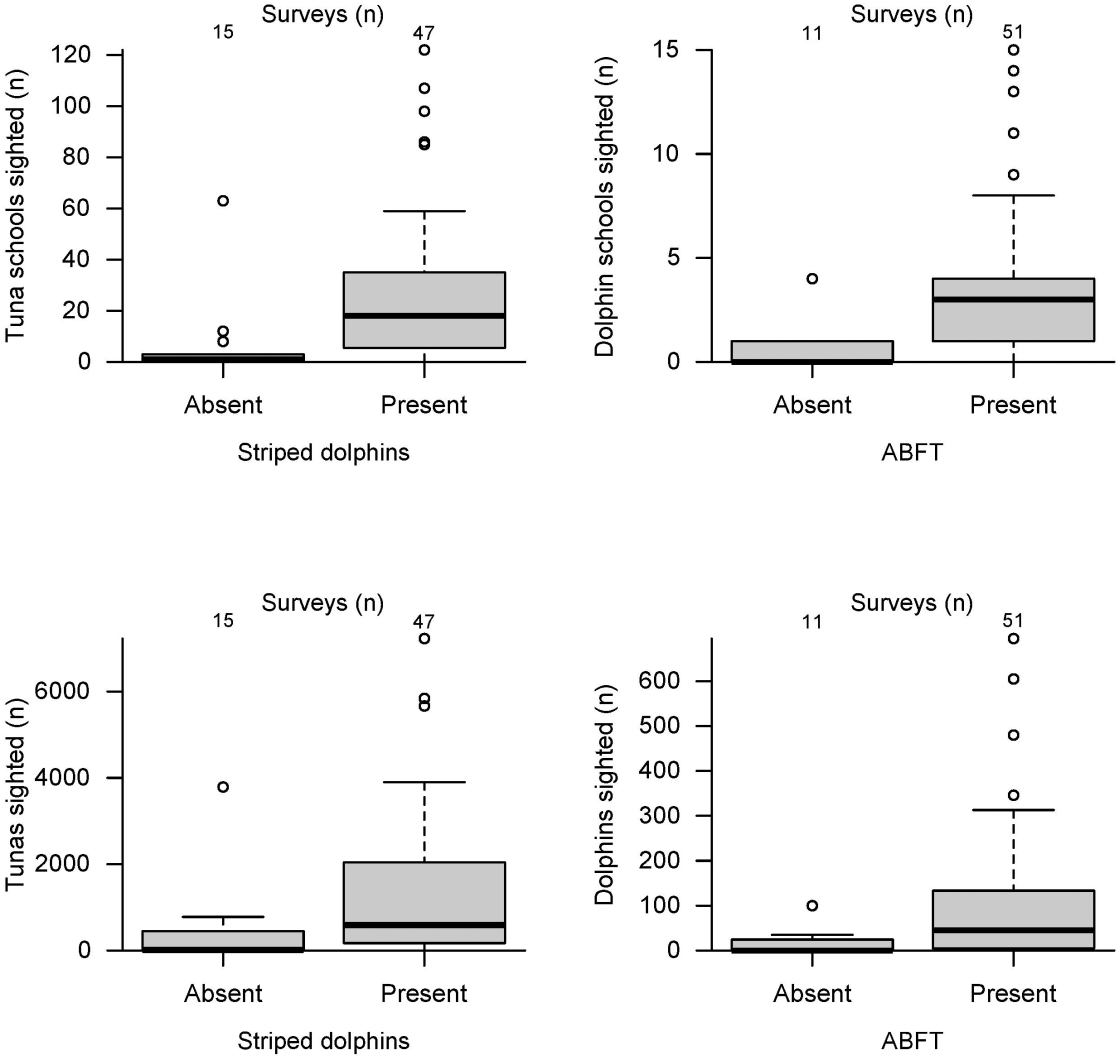
Number of sighted schools (Upper panel) and individuals (lower panel) for ABFT (left column) and striped dolphins (right column) per aerial survey dependent on the absence/presence of the other species. Note that the number of sighted tunas was recalculated from school size estimates based on purse seine catch data. See Bauer et al. [11] for more details.

ABFT was the most frequently sighted species during all survey years. Each year 48 to 267 sighting events included surface feeding tunas, often in multiple schools. The number of sighted ABFT was significantly higher when dolphins were present in the survey area (Fig 2), and consequently, the same trend was observed for striped dolphins.

### Detectability and line transect modeling

**Fig 3:**
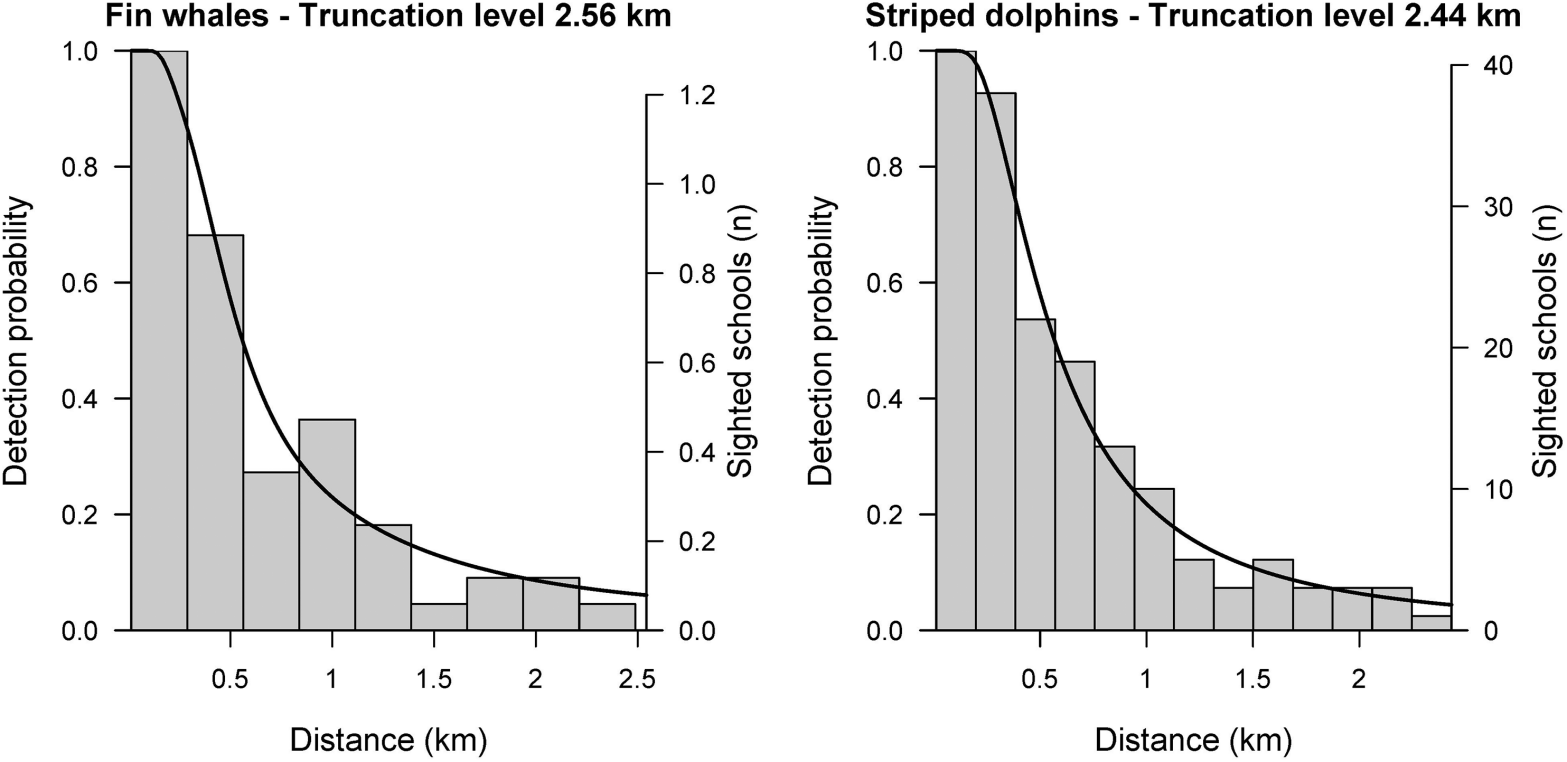
Detection functions (black line) of best line transect model fits for fin whale (left) and striped dolphin (right) sighting data (grey), averaged over estimated covariate levels.

For fin whales and striped dolphins, best model fits, selected by the Akaike’s information criterion (AIC), were obtained from the multiple-covariate approach based on a hazard rate key-function (Fig 3). All goodness of fit tests (Cramer-von Mises and Kolmogorov-Smirnov tests) performed well for the selected models of both species (p > 0.05). No significant deviations were evident in the q-q plot of the striped dolphin model, and only slight deviations in case of the fin whale model, which could relate to the small sample size. Model results indicated significant effects on detectability due to the sea state for both cetacean species and in the case of dolphins the number of observers also showed an effect. No effect related to the observer team, the plane used or the school size was found for both fin whales or striped dolphins. However, model fits for ABFT also included effects by the observer team and school size. For more details on ABFT model fits please refer to Bauer et al. [11].

### Availability bias

Based on the aircraft speed, we estimated an availability probability of 0.672 for small striped dolphin schools (≦ 15 individuals). For larger schools availability was assumed to be certain, in accordance with observations made by Gómez De Segura et al. [33]. The availability probability *a*(*S*, 0) for fin whales was estimated as 0.245 (bootstrapped *CV* = 0.53). As stated earlier, no such estimate could be obtained for fin whale schools, due to the lack of respective diving data. This may imply that fin whale density estimates are negatively biased. However, we assume this effect to be of minor importance, given the small average school size of fin whales. In the case of fin whales, availability estimates indicate that a high proportion of fin whales were missed during the aerial surveys, since fin whales spend more time diving than at the surface.

### Sighting rates and density estimates

**Fig 4:**
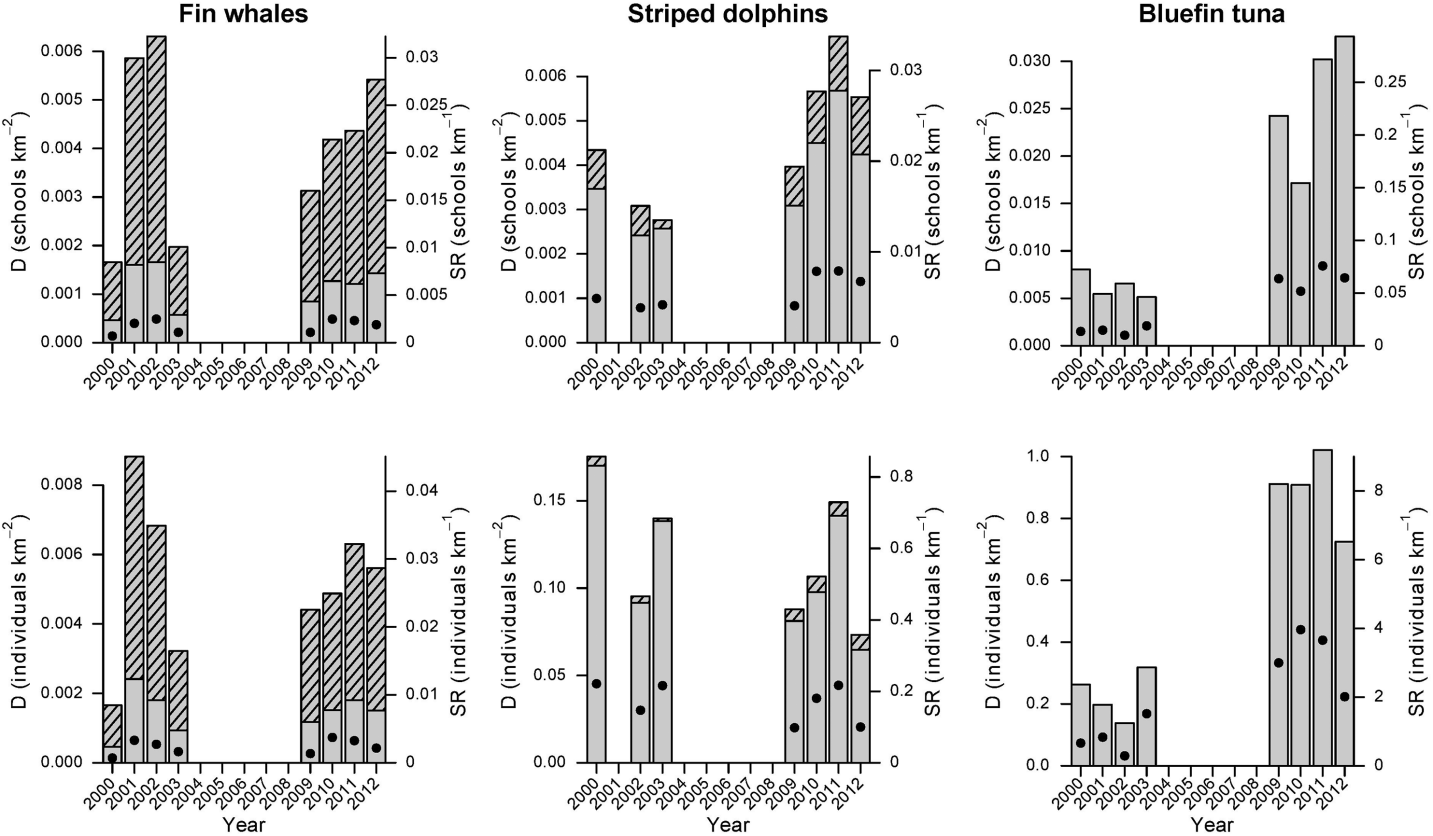
Sighting rates (dots), uncorrected densities (grey bars) and density estimates corrected for availability bias (grey bars with shaded area) of schools (upper) and individuals (lower panel) of fin whales (left), striped dolphins (center) and blue fin tunas (right) in the GoL, derived from the line transect approach (5% right truncation for striped dolphins and tunas, 10% for fin whales). Note that SR and density estimates are proportional, i.e. density estimates can be projected on the SR axis, indicating the total number of sightings possible within the truncation width. Vise versa, SR estimates correspond to strip transect density estimates if all sightings were made within the truncation width (and are therefore 5–10% lower than true values, depending on the truncation level). For precise SR and density estimates, see Tables 2–4.

Sighting rates (SR) and density estimates for both cetacean species (uncorrected and corrected for availability bias) as well as ABFT blackare given in Fig 4 (for precise estimates see Tables 2–4). Note that density estimates of ABFT differ slightly from those in Bauer et al. [11] due to methodological adjustments in the density estimation process, although the same fitted detection functions were applied (e.g. to increase precision, all replicates of one year were now pooled together, instead of calculating the average across replicates). Sighting rates were lowest for fin whales with 0.0007–0.0017 schools and 0.0007–0.0037 individuals detected per km. Dolphin schools were more frequently detected with sighting rates in the range of 0.0038–0.0079 schools per km and 0.1000–0.2211 individuals per km. Most detections were made for ABFT (0.0099–0.0757 schools per km and 0.2895–3.9633 individuals per km). Uncorrected density estimates were generally proportional to sighting rates, but 3–5 times higher. Availability bias had considerable effects on school and absolute densities of fin whales, with corrected estimates being three times higher than uncorrected values. Differences between corrected and uncorrected estimates of striped dolphin school densities were much less pronounced. Absolute densities of striped dolphin were almost unaffected by availability bias, as only small dolphin schools were assumed to be affected by availability bias which contributed little to overall densities. In fact, corrected school densities of fin whales and striped dolphins are of the same magnitude, ranging between 0.002 and 0.006 schools per km^2^. However, given the small school sizes of fin whales (Table 2), absolute densities of fin whales were lowest among the studied species and furthermore remained comparable during both survey periods (2000–2003 and 2009–2012). Nonetheless, fin whale densities showed significant year-to-year variations without any trend, with lower densities in 2000 and 2009 and higher densities in 2001 and 2010. By contrast, school densities (and thus sighting numbers) of striped dolphins and ABFT were significantly higher during 2009–2012. This pattern remained evident for absolute ABFT densities (number of individuals), but not for striped dolphins, due to smaller dolphin schools observed during this period (Table 3). Note that ABFT school size also decreased during the second survey period, notably during the 2011 and 2012 (Table 4; [11]), in accordance with the increase of ABFT densities during the latter years. Density estimates of all species varied between survey replicates (data not shown here). This applies particularly to fin whales, as a consequence of relatively low sighting numbers, i.e. a high number of surveys without sightings.

**Table 2:** Fin whales sighting rates (SR; n/km) as well as density estimates (D, n/km^2^) for numbers of schools and individuals with corresponding coefficients of variation (%CV) and 95% confidence intervals (CI). Estimates corrected for availability bias are given in parentheses.

Year	Schools				Mean school size	Individuals			
	SR	D	%CV	95% CI		SR	D	%CV	95% CI
2000	0.0007	0.0005 (0.0017)	72.9 (67.6)	0.0001-0.0021 (0.0003-0.0079)	1.0	0.0007	0.0005 (0.0017)	72.9 (67.6)	0.0001-0.0021 (0.0003-0.0079)
2001	0.0020	0.0016 (0.0059)	42.2 (32.8)	0.0007-0.0037 (0.0028-0.0123)	1.6	0.0033	0.0024 (0.0088)	47.6 (43.7)	0.0009-0.0065 (0.0033-0.0235)
2002	0.0025	0.0017 (0.0063)	49.5 (46.0)	0.0006-0.0046 (0.0023-0.0173)	1.1	0.0027	0.0018 (0.0068)	48.3 (44.7)	0.0007-0.0049 (0.0026-0.0182)
2003	0.0011	0.0006 (0.0020)	67.6 (64.6)	0.0002-0.0022 (0.0005-0.0073)	1.5	0.0016	0.0009 (0.0032)	80.0 (76.8)	0.0002-0.0043 (0.0007-0.0146)
2009	0.0011	0.0008 (0.0031)	73.8 (60.5)	0.0002-0.0034 (0.0009-0.0115)	1.2	0.0014	0.0012 (0.0044)	75.5 (63.1)	0.0003-0.0049 (0.0011-0.0170)
2010	0.0025	0.0013 (0.0042)	52.8 (48.7)	0.0004-0.0039 (0.0013-0.0134)	1.5	0.0037	0.0015 (0.0049)	49.6 (45.3)	0.0005-0.0044 (0.0016-0.0145)
2011	0.0023	0.0012 (0.0044)	74.8 (71.8)	0.0003-0.0055 (0.0010-0.0193)	1.4	0.0033	0.0018 (0.0063)	83.7 (80.4)	0.0003-0.0094 (0.0012-0.0321)
2012	0.0019	0.0014 (0.0054)	34.1 (24.7)	0.0007-0.0029 (0.0029-0.0103)	1.1	0.0022	0.0015 (0.0056)	34.3 (25.4)	0.0007-0.0031 (0.0029-0.0109)

**Table 3:** Striped dolphin sighting rates (SR; n/km) as well as density estimates (D, n/km^2^) for numbers of schools and individuals with corresponding coefficients of variation (%CV) and 95% confidence intervals (CI). Estimates corrected for availability bias are given in parentheses.

Year	Schools				Mean school size	Individuals			
	SR	D	%CV	95% CI		SR	D	%CV	95% CI
2000	0.0049	0.0035 (0.0043)	41.5 (42.1)	0.0013-0.0091 (0.0016-0.0118)	45.4	0.2211	0.1701 (0.1753)	81.0 (78.0)	0.0284-1.0198 (0.0308-0.9975)
2001	-	-	-	-	-	-	-	-	-
2002	0.0038	0.0024 (0.0031)	60.1 (59.7)	0.0007-0.0085 (0.0009-0.0108)	38.2	0.1469	0.0916 (0.0953)	71.5 (70.8)	0.0213-0.3946 (0.0224-0.4064)
2003	0.0042	0.0026 (0.0028)	35.6 (34.5)	0.0012-0.0054 (0.0013-0.0057)	51.7	0.2159	0.1383 (0.1398)	44.0 (43.6)	0.0557-0.3439 (0.0567-0.3450)
2009	0.0041	0.0031 (0.0040)	49.8 (47.3)	0.0011-0.0091 (0.0014-0.0112)	24.1	0.0980	0.0812 (0.0878)	80.5 (77.3)	0.0156-0.4212 (0.0178-0.4329)
2010	0.0079	0.0045 (0.0057)	49.9 (53.6)	0.0014-0.0144 (0.0016-0.0200)	22.9	0.1804	0.0977 (0.1066)	30.0 (31.4)	0.0497-0.1922 (0.0518-0.2197)
2011	0.0079	0.0057 (0.0069)	34.1 (33.4)	0.0027-0.0118 (0.0033-0.0143)	27.4	0.2167	0.1415 (0.1492)	33.8 (32.4)	0.0679-0.2947 (0.0736-0.3023)
2012	0.0067	0.0042 (0.0055)	45.3 (44.0)	0.0014-0.0128 (0.0018-0.0168)	14.8	0.1000	0.0647 (0.0732)	46.3 (45.1)	0.0207-0.2022 (0.0237-0.2258)

**Table 4:** Bluefin tuna sighting rates (SR; n/km) as well as density estimates (D, n/km^2^) for numbers of schools and individuals with corresponding coefficients of variation (%CV) and 95% confidence intervals (CI).

Year	Schools				Mean school size	Individuals			
	SR	D	%CV	95% CI		SR	D	%CV	95% CI
2000	0.0136	0.0080	42.6	0.0031-0.0210	59.4	0.6611	0.2624	36.8	0.1163-0.5922
2001	0.0147	0.0055	50.9	0.0018-0.0167	72.8	0.8332	0.1974	43.9	0.0746-0.5227
2002	0.0099	0.0065	85.0	0.0012-0.0349	32.8	0.2895	0.1378	74.6	0.0305-0.6220
2003	0.0189	0.0051	34.6	0.0025-0.0107	100.7	1.5165	0.3186	31.1	0.1651-0.6148
2009	0.0636	0.0242	39.5	0.0101-0.0582	75.8	2.9974	0.9112	47.6	0.3191-2.6022
2010	0.0517	0.0172	69.9	0.0035-0.0852	171.1	3.9633	0.9083	75.2	0.1647-5.0081
2011	0.0757	0.0302	32.3	0.0149-0.0613	83.3	3.6554	1.0211	28.0	0.5511-1.8919
2012	0.0644	0.0326	26.3	0.0169-0.0630	32.2	2.0111	0.7248	31.4	0.3250-1.6164

### Spatial distribution and co-occurrence

**Fig 5:**
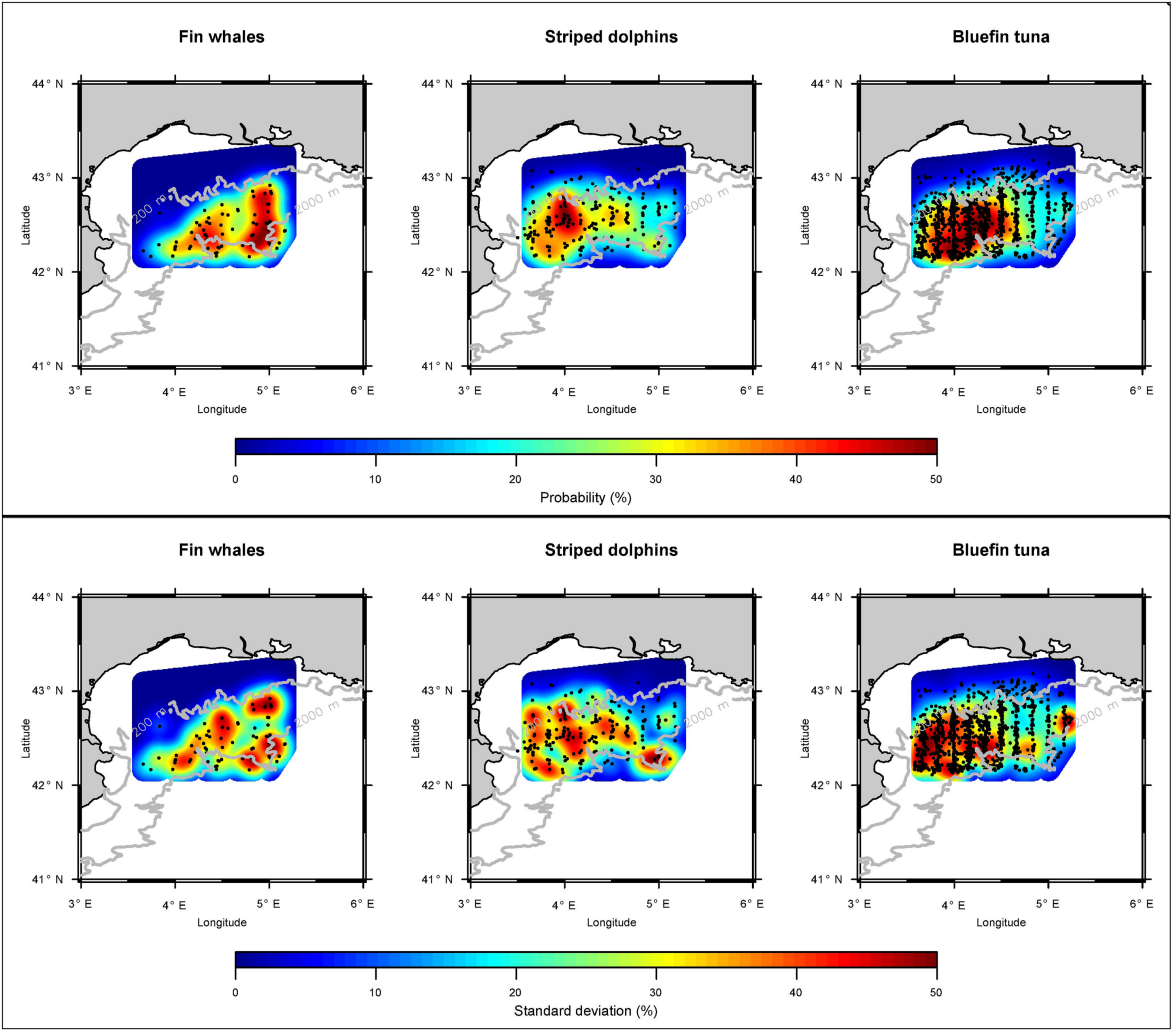
Average sighting location probabilities (top panel) and standard deviation (lower panel) of fin whales (left), striped dolphins (center) and ABFT (right). Actual sighting locations are indicated by black dots. The dashed lines (grey) represent the 200 and 2000 m isobaths, indicating the continental shelf break of the Gulf of Lions.

As with ABFT, fin whales and striped dolphins were most frequently sighted on the shelf break area of the survey region, between the 200 and 2000 m depth contours (Fig 5). However, the spatial distributions of the three species showed limited consistency in the overlap of core density areas (Table 5). This is especially true for ABFT and fin whales, while ABFT and striped dolphins overlapped more regularly. Fin whales were mostly found in the central and eastern part of the surveyed shelf break, dolphins and ABFT were most frequently sighted in the western area. In addition, sightings of striped dolphins on the shelf area were not uncommon, where fin whales were practically absent. A closer link between sighting locations of striped dolphins and ABFT is also evident from the co-occurrence indices (Table 6). Accordingly, 23.9% of sighted striped dolphins schools were located in the proximity of ABFT. Remarkably, this relationship was absent for the proximity of ABFT relative to striped dolphins. The co-occurrence between fin whales and both of these species appears to be less pronounced.

**Table 5:** Overlap of core areas (50% kernel density) of fin whales, striped dolphins and ABFT per survey year in percent.

Survey year	Fin whales & striped dolphins	Fin whales & ABFT	Striped dolphins & ABFT
2000	0	0	0
2001	-	0	-
2002	0	18.3	4.7
2003	0.1	29.9	25.1
2009	0.6	0.8	3.2
2010	11.2	3.2	8.3
2011	0	0	16.4
2012	0	3.3	15.3

**Table 6:** Co-occurrence of fin whales, striped dolphins and ABFT during the aerial surveys as percentage of sighting positions within a radius of 2.5 km.

Species	Sighting positions					
	Total (n)	Alone (%)	With fin whales (%)	With striped dolphins (%)	With ABFT (%)	With both other species (%)
Fin whales	70	77.1	-	7.1	12.9	2.9
Striped dolphins	180	73.3	2.8	-	22.8	1.1
ABFT	1022	91.9	1.2	6.6	-	0.3

### Ocean fronts

**Fig 6:**
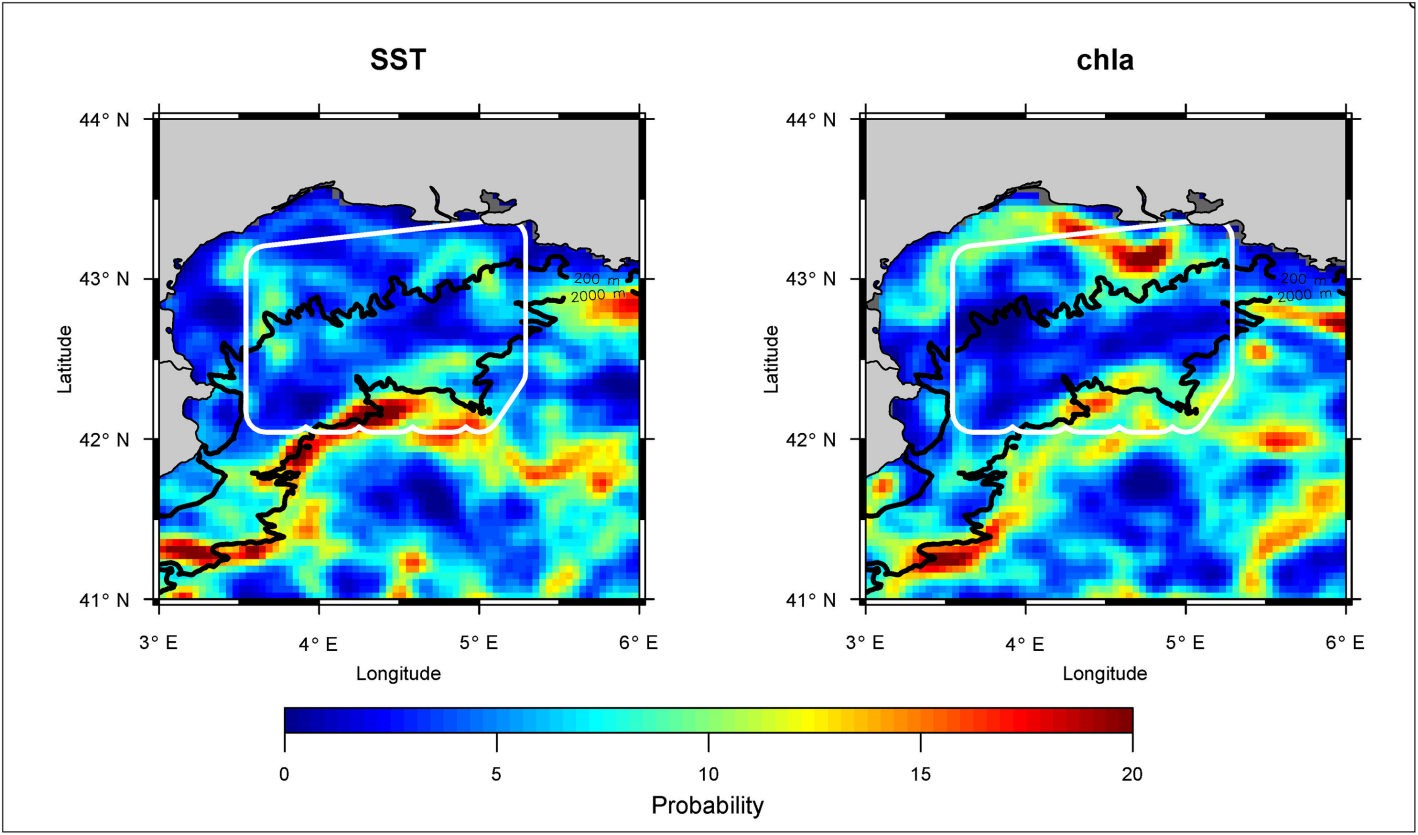
Average positions of SST (left) and chla (right) fronts during the survey dates since 2002. The dashed lines represent the 200 and 2000 m isobaths, indicating the continental shelf break of the Gulf of Lions. The survey area is circled in white.

Major ocean front structures during the survey period, derived from SST and chla satellite images, were detected along the 2000 m isobar, in the central and south-western part of the GoL (Fig 6). These structures coincide with persistent flow positions of the Northern Current [43]. Strong chla front structures are further evident on the continental shelf of the GoL that are linked to the influx of nutrient-rich runoff from the Rhone river.

## Discussion

In this study, we present and compare results of densities and sighting distribution patterns of three top predator species, fin whales, striped dolphins and ABFT. Presented results are derived from line transect modeling based on data from eight years of aerial surveys, conducted in the GoL, Northwestern Mediterranean Sea. This area is an important feeding ground of ABFT and both cetacean species, particularly during the major feeding season of fin whales and ABFT (August—October). Results indicate that density, presence and core sighting locations of striped dolphins and ABFT are correlated, although their feeding habits differ (e.g. their prey, foraging depths and diurnal activity patterns). Core sighting areas of these species were located close to an area of high mesoscale activity (oceanic fronts and eddies). Fin whales were less abundant and did not show a similar correlation. The study demonstrates an example for the successful application of aerial surveys for monitoring multi-species abundance.

### Detectability

Unlike ABFT, fin whales and striped dolphins were seldom sighted while feeding at the surface. Striped dolphins were frequently seen traveling. As a consequence, their “cue production” was found to be less detectable from afar than surface feeding ABFT. Half of the fin whale and dolphin detections were made at distances of less than 0.5 km from the transect line which is in accordance with results from other aerial surveys. By contrast, the very distinct and more localized water disturbance produced by large tuna schools feeding at the surface (“breezer” and “jumpers”; [44]) is visible from several km [11]. Moreover, unlike ABFT, school size did not affect the detectability of dolphins or fin whales. No effect on the detectability was apparent from the change in plane and the related altitude change. This may indicate that dolphins and fin whales can still be quantitatively detected at the higher altitude of 1500 feet used in 2012. However, the low sample sizes for both species make it difficult to identify factors influencing detectability, that can therefore not be fully excluded.

### Availability and perception bias

Availability bias was found to be particularly important for fin whales as this species spent only a small proportion of their time at the surface. As a consequence, only few fin whales were detected, which makes the corresponding density estimates uncertain. In fact, annual fluctuations in fin whale availability, that could not be assessed here, could largely affect sighting numbers and thus density estimates, Although fin whales are obliged to surface regularly, such fluctuations are possible, e.g. due to changes in the depth distribution and patchiness of prey organisms that may alter feeding depths, as well as the frequency and duration of dives. Such effects seem to be less likely for striped dolphins with regards to their higher availability estimates and larger school sizes, as multiple individuals need to dive simultaneously to be overlooked. However, surveys without detections were not uncommon for all the species investigated, although sighting conditions were perfect and the species were detected shortly afterwards in subsequent surveys. In the case of ABFT, such events are particularly striking given the high numbers of schools regularly being detected. Accordingly, the species may have completely left the survey area for a short time or just shifted to deeper waters, while still being present. Considering the high mobility of the species studied ([44; 46] with references therein), both scenarios are conceivable, further highlighting the need to evaluate the surface availability of these species and the processes that influence it. In this regard, our results on the co-occurrence of the three species provide first indications of these processes (see below), while availability estimates give an impression of the potential impact of availability on related density estimates of the two cetacean species.

As stated earlier, density estimates of this study could not be corrected for perception bias (i.e. accounting for animals that were missed by the observers, although they were available for detection on the transect line). Despite the experience of our observers, such an effect can not be excluded, especially since the aircrafts used were not equipped with bubble windows that provide better visibility of the area beneath the aircraft. However, detection frequencies for any species did not suggest that animals were systematically missed on the transect line. Further sources of perception bias include the speed and operational altitude, which will be further discussed below (see subsection “Towards multi-species aerial surveys”). Results from other studies indicate that the perception probability is usually high and depends on the school size (e.g. 1 for large schools of dolphins and 0.86 for fin whales at west Greenland; [47]). Overall density estimates might therefore be more influenced by detectability and availability.

### Density, spatial distribution and co-occurrence

During this study, fin whales were infrequently sighted and seldom in groups. The average group consisted of 1.33 individuals (95% CI 1.19–1.47) which is in the lower range of literature values from the Atlantic Ocean(1.4–2.5 individuals) and the Northwestern Mediterranean Sea (1.44 individuals, [48]; Table A in S1 File). Although fin whales are assumed to be more concentrated on the feeding grounds during summer and autumn [48], lower group size might reflect dispersed food availability, thus reducing food competition [49]. In accordance to this observation, fin whale density estimates were in the lower range of literature estimates from the Northwestern Mediterranean Sea (Fig 4; Table 2 and Table A in S1 File). This could further reflect habitat preferences, as in the Northwestern Mediterranean Sea fin whales are reportedly more frequently found in deeper off-shore waters [48; 50], although feeding habitat predictions suggest a much higher importance of the canyon-rich GoL [51]. In fact, our survey effort was centered on the continental shelf break while most previous studies cover large off-shore areas (Table A in S1 File). The high variability in annual fin whales density estimates supports the theory that the distribution of plankton-feeding fin whales in the Northwestern Mediterranean Sea is less predictable than those of ichthyophagous populations [52].

Striped dolphins are also considered to prefer deeper waters [53; 54]. Here, striped dolphins were sighted in waters with an average depth of 1123 m (range: 79–2180 m), while the surveyed area had an average depth of 967 m (Fig 6). Accordingly, density estimates for striped dolphins were likewise comparably low (Fig 4; Table 3 and Table B in S1 File), but of the same magnitude as estimates from shelf break areas of the Ligurian Sea (Pelagos Sanctuary), which is located to the Northeast of our study region (e.g. 0.1486–0.1859 dolphins/km^2^ reported by Panigada et al. [55]).

In fact, fin whales and striped dolphins were most frequently sighted on the shelf break area of the survey region and seldom on the shelf area. Gannier [53] showed that in the Ligurian Sea, striped dolphins feed nearshore during the night and then migrate offshore in the morning, a behaviour that may explain our infrequent dolphin sightings on the shelf. In fact, striped dolphins were almost exclusively seen when not feeding and not associating with the usually 3–10 times more abundant ABFT or the less common fin whales. However, dolphins frequently co-occurred with ABFT, although not vice-versa. It may hence be hypothesized that striped dolphins could be attracted by the feeding activity of ABFT, as yearly core sighting areas of both species also showed some overlap in the southwestern part of the GoL. Both species are opportunistic predators [56], with ABFT feeding mainly in the epipelagic zone of the GoL and by daylight, on small pelagic species such as anchovies and sardines, and to a lesser extent on squid. Stomach contents of stranded animals in the Northwestern Mediterranean showed that striped dolphins feed to a much higher degree on squid (ca. 50% of food biomass), but also occasionally on small pelagic fish [57; 58; 59]. For example, Würtz et al. [57] found anchovies in the stomachs of two of 23 stranded dolphins (8.7%). However, no such data is available for the GoL, impeding a more detailed comparison.

As the occurrence and distribution of dolphins and ABFT in the survey zone appears to be slightly linked, their presence (and/or surface availability) may depend on the same factors. Such factors may trigger the productivity of the entire survey zone, such as its mesoscale activity or inactivity (e.g. fronts and eddies). In fact, the southwestern part of the GoL, where core areas of ABFT and striped dolphins overlap, is characterized by strong currents and is a known building zone of mesoscale eddies [60; 61]. Accordingly, the Ligurian-Provencal front appeared to be strongest close to this area during the survey period.

By contrast, core areas of fin whales were situated to the central and eastern part of the shelf break area, which is similar to the findings of a previous analysis from other sighting from 1993–2001 by Monestiez et al. [62]. This area is influenced more strongly by the Rhone runoff and characterized by multiple canyons that significantly contribute to the water exchange between the shelf waters of the GoL and the open sea [60]. In fact, such canyon-rich areas are known to attract fin whales as they often host higher aggregations of mesopelagic krill (*Meganyctiphanes norvegica*), the primary prey of fin whales in the Northwestern Mediterranean Sea [63; 64; 48]. As with striped dolphins, fin whales may also occasionally feed on sardines and anchovies in the GoL [65]. However, they are seldom observed feeding at the surface [48], as observed in this study. While in the present study no evidence was found that habitat selection of fin whales and juvenile ABFT are linked, tagging results on mature ABFT and fin whales in the Northwestern Mediterranean indicate a strong overlap in their respective core-areas, which are located just to the south of the survey region, between the Northern current and the Balearic front [66; 67]. It would therefore be very interesting to extend the aerial survey region further towards this area.

### Towards multi-species aerial surveys

Apart from cetaceans, aerial surveys are applied to assess the density and abundance of a variety of species, including sea turtles, elasmobranchs and tuna [68; 69]. As shown in this study, the frequent sightings of non-target species, made during the surveys, may allow the calculation of multi-species abundance indices and thus provide crucial knowledge for their conservation. Such an opportunity is of great interest for the implication of conservation policies, in particular for the development of monitoring strategies as it can help to coordinate efforts. In fact, such a development has been requested under the Marine Strategy Framework Directive (MSFD). However, the ability to quantitatively detect multiple species during an aerial survey depends on the survey design, mainly on the requirements to detect the target species. This particularly concerns the operational altitude and traveling speed at which the surveys are conducted. Higher traveling speeds decrease the time to identify objects, in particular at lower altitudes. Lower altitudes, however, facilitate the identification of smaller species and single animals (e.g. turtles) which is essential for the abundance estimation of very rare species. Nonetheless, higher altitudes increase the detectability and availability probability of more distant schools and thus the spatial representativeness of the surveys. As such, higher altitudes might be more suitable for tunas, as large aggregation zones remain detectable at distances of more than 10 km [11]. Both the selection of altitude and traveling speed therefore represent an important source for the perception bias [70; 71]. Although aerial surveys on marine mammals are conducted at a wide range of altitudes (600–1500 ft; see references in Rowat et al. [68]), very few studies have tried to assess the actual effects of the altitude. Related approaches usually include mark-recapture distance sampling [70]. Apart from this, Laake et al. [71] provide an example how the variation of altitudes during an aerial survey can be incorporated in the modeling process of the detection function. While such altitude effects should not be neglected, particularly in the survey design, Gosselin et al. [72] suggested, that such effects are probably minor in comparison to variations in daily abundance, based on a comparative analysis between aerial surveys conducted at 1000 and 1500 ft on beluga whales (*Delphinapterus leucas*) in the St Lawrence Estuary. The results of the present study indicate that densities of all three species evaluated from the survey data, ABFT, fin whales and striped dolphins, can be accurately assessed at the selected altitudes. The comparable literature values on cetacean densities from aerial surveys conducted at lower depths in areas close to the study region support this assumption (Tables A and B in S1 File).

## Supporting Information

S1 File Figures and tables. Table A, Literature values on fin whale sighting rates (per km) and density (per km^2^) estimates from the western Mediterranean Sea. Table B, Literature values on striped dolphin sighting rates (per km) and density (per km^2^) estimates from the western Mediterranean Sea. Fig A, Dolphin school size in number of individuals per survey year.
(PDF)

S2 File Zip archive with aerial survey data. (ZIP)
