# Supplementary material for: Co-Occurrence and Habitat Use of Fin Whales, Striped Dolphins and Atlantic Bluefin Tuna in the Northwestern Mediterranean Sea
Source: PLoS One. 2015 Oct 12;10(10):e0139218. doi: 10.1371/journal.pone.0139218 (PMC4601798; doi:10.1371/journal.pone.0139218)
Supplement: S1 File — Table A, Literature values on fin whale sighting rates (per km) and density (per km2) estimates from the western Mediterranean Sea. Table B, Literature values on striped dolphin sighting rates (per km) and density (per km2) estimates from the western Mediterranean Sea. Fig A, Dolphin school size in number of individuals per survey year. (PDF) [file pone.0139218.s001.pdf]

Co-occurrence and habitat use of fin whales, striped dolphins and Atlantic bluefin tuna in the Northwestern Mediterranean Sea

Robert Klaus Bauer[1], Jean-Marc Fromentin[1¶], Hervé Demarcq[2], Blandine Brisset[1] and Sylvain Bonhommeau[1¶]

[1]IFREMER (Institut Français de Recherche pour l'Exploitation de la MER), Resarch Unit Marbec (Marine Biodiversity, Exploitation and Conservation), Sète, France
[2]IRD (Institut de Recherche pour le Développement), Resarch Unit Marbec (Marine Biodiversity, Exploitation and Conservation), Sète, France

*Corresponding author:
Email: *robert.bauer@ifremer.fr* (RKB)

¶These authors contributed equally to this work.

Supporting material

Table A Literature values on fin whale sighting rates (per km) and density (per km^2) estimates from the western Mediterranean Sea, all uncorrected for availability bias. (* Total study area)

Study	Survey years	Period	Region	Platform	Effort	SR schools	SR	School size	D
Forcada et al. [1]	1991	August	Western Mediterranean	shipboard	6852	0.063		1.44	0.024
Forcada et al. [2]	1992	August	Western Ligurian Sea	shipboard	1151.8	0.041		1.5	0.016
Gannier [3]	1991-1994	July–August	Provencal shelf	shipboard			0.011		0.009
			Ligurian Sea				0.021		0.017
			Corsican shelf				0.013		0.011
			Ligurian-Provencal Basin*		4400			1.53	0.015
Gannier [4]	1998	winter	Ligurian Sea	shipboard			0.003		
		spring					0.005		
		summer (June–September)					0.041		
Gannier et al. [5]	1998	August	Gulf of Lions	shipboard	405.6		0.002	1.4*	
			Ligurian Sea		401.9		0.013		
Laran et al. [6]	2001-2004	April	Ligurian Sea	shipboard	5759		0.026	1.5 (1.3-1.9)	
		July-August					0.044-0.056		
Laran et al. [7]	2001-2004	Winter (October–March)	Ligurian-Provencal	shipboard	2235			1.1	0.002
		Summer (April–September)			3967			1.6	0.014
Lauriano et al. [8]	2008	August	Ligurian-Provencal	shipboard	1255	0.010		1.08	
Panigada et al. [9]	2009	Winter	Ligurian Sea	plane	8144.5	0.0001	0.0001	1	
		Summer			8446.9	0.002	0.002	1	0.002

Table B Literature values on striped dolphin sighting rates (per km) and density (per km^2) estimates from the western Mediterranean Sea, all uncorrected for availability bias, except estimates from Gómez De Segura et al. [10]. (* Total study area)

Study	Survey Years	Period	Region	Platform	Effort	SR schools	SR	School size	D
Forcada et al. [11]	1991	August–September	Western Mediterranean	shipboard	6852	0.024		19.7	0.20
Forcada et al. [12]	1991	August–September	Balearic Sea	shipboard	6852		0.014	17.2	0.09
			Provencal basin				0.034	13.0	0.23
			Ligurian Sea				0.042	14.2	0.30
			Alboran Sea				0.014	42.8	0.20
			South Balearic				0.016	10.1	0.08
	1992	June	Alboran Sea		992		0.009	20.2	
Forcada et al. [2]	1992	August	Western Ligurian Sea	shipboard	1151.8	0.058		22.5	0.44
Gannier [13]	1996	Summer	Ligurian Sea	shipboard	1398,3		0.04	16.4	0.56
Gannier [14]	1989-1997		Ligurian Sea (Near-Shore)	shipboard	1267		1.46	18.1	
			Ligurian Sea (Slope)		1793		0.73	16.7	
			Ligurian Sea (Offshore)		1151		1.43	22.1	
			Ligurian Sea (Open Sea)		858		2.32	22.2	
Gannier [4]	1998		Ligurian Sea	shipboard			0.79		
Gannier et al. [5]	1998	August	Gulf of Lions	shipboard	405.6		0.35	19.5*	
			Ligurian Sea		401.9		0.67		
Gannier [15]	1997-2001		NW Mediterranean	shipboard	4824	0.0324	0.76	23.3	
			SW Mediterranean		2156	0.0187	0.37	19.8	
			Alboran Sea		1065	0.0366	1.11	30.2	
			NW Tyrrhenian Sea		663	0.0181	0.53	29.2	
			SW Tyrrhenian Sea		1583	0.0158	0.40	25.4	
			Ionian Sea		2576	0.0109	0.33	30.7	
			Levantine Basin		580	0.0107	0.14	13.3	
Fortuna et al. [16]	2002	July	Southern Tyrrhenian Sea	shipboard	542		0.04	15.1	0.26
	2003	May			1011				0.30
Gómez De Segura et al. [10]	2001–2003	total period (May–March)	Western Mediteranean Sea (Spain)	plane	16754				0.489
		Winter (January–March)			4190			10	0.580
		Spring (April–June)			4302			32	0.334
		Summer (July–September)			3955			21	0.522
		Autumn (October–December)			4306			14	0.600
Laran et al. [6]	2001-2004	December–April	Ligurian-Provencal	shipboard	5759*		0.18-0.66	18.0	
		May/September					1.34/1.52		
Laran et al. [7]	2001-2004	Winter (October–March)	Ligurian-Provencal	shipboard	2235			10.9	0.37
		Summer (April–September)			3967			19.9	0.87
Lauriano et al. [8]	2008	August	Ligurian-Provencal	shipboard	1255		0.026	7.51	0.228
Panigada et al. [9]	2009	Winter	Ligurian Sea	plane	8144.5		0.01	8	0.22
		Summer			8446.9		0.03	13.7	0.436

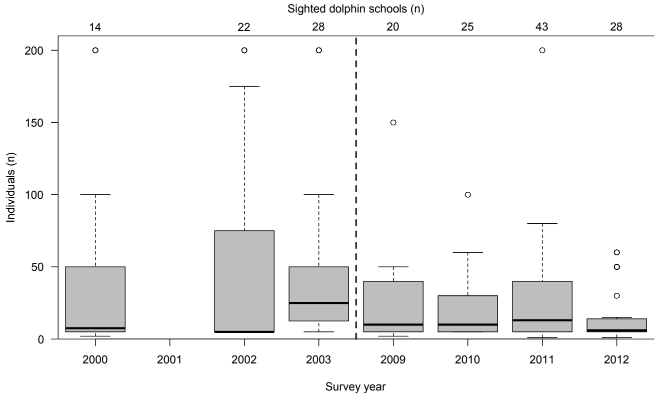

Fig A Dolphin school size in number of individuals per survey repetition and year.

References

[1] Forcada J, Aguilar A, Hammond P, Pastor X, Aguilar R. Distribution and abundance of fin whales (*Balaenoptera physalus*) in the western Mediterranean sea during the summer. J Zool. 1996;238(1):23–34.

[2] Forcada J, Notarbartolo Di Sciara G, Fabbri F. Abundance of fin whales and striped dolphins summering in the Corso-Ligurian Basin. Mammalia. 1995;59(1):127–140.

[3] Gannier A. Estimation de l'abondance estivale du rorqual commun *Balaenoptera physalus* (Linné, 1758) dans le bassin liguro-provençal (Méditerranée occidentale). Rev d'écologie (Terre Vie). 1997 Dec;52(1):69–86.

[4] Gannier A. Les cétacés de Méditerranée nord-occidentale : nouveaux résultats sur leur distribution, la structure de leur peuplement et l'abondance relative des différentes espèces. Mésogée. 1999;56:3–19.

[5] Gannier A, Bourreau S. Comparison of cetacean populations from simultaneous surveys in the Gulf of Lion and Ligurian areas. Eur Res Cetaceans. 1999;13.

[6] Laran S, Drouot-Dulau V. Seasonal variation of striped dolphins, fin- and sperm whales' abundance in the Ligurian Sea (Mediterranean Sea). J Mar Biol Assoc UK. 2007 Feb;87(01):345.

[7] Laran S, Joiris C, Gannier A, Kenney R. Seasonal estimates of densities and predation rates of cetaceans in the Ligurian Sea, northwestern Mediterranean Sea: an initial examination. J Cetacean Res Manag. 2010;11(1):31–40.

[8] Lauriano G, Panigada S, Canneri R, Zeichen MM. Abundance estimate of striped dolphins (*Stenella coeruleoalba*) in the Pelagos sanctuary (NW Mediterranean) by means of line transect surveys. J Cetacean Res Manag. 2010;11(3):279–284.

[9] Panigada S, Lauriano G, Burt L, Pierantonio N, Donovan G. Monitoring Winter and Summer Abundance of Cetaceans in the Pelagos Sanctuary (Northwestern Mediterranean Sea) Through Aerial Surveys. PLoS One. 2011;6(7):e22878.

[10] Gómez De Segura A, Crespo Ea, Pedraza SN, Hammond PS, Raga Ja. Abundance of small cetaceans in waters of the central Spanish Mediterranean. Mar Biol. 2006;150(1):149–160.

[11] Forcada J, Aguilar A, Hammond PS, Pastor X, Aguilar R. Distribution andnumbers of striped dolphins in the western Mediterranean after the 1990 epizootic outbreak. Mar Mammal Sci. 1994;10(2):137–150.

[12] Forcada J, Hammond P. Geographical variation in abundance of striped and common dolphins of the western Mediterranean. J Sea Res. 1998;39(3-4):313–325.

[13] Gannier A. Une estimation de l'abondance estivale du dauphin bleu et blanc *Stenella coeruleoalba* (Meyen, 1833) dans le futur sanctuaire marin international de mediterranee nord-occidentale. Rev d'Ecologie (La Terre la Vie). 1998;53(3):255–272.

[14] Gannier A. Diel variations of the striped dolphin distribution off the French Riviera (Northwestern Mediterranean Sea). Aquat Mamm. 1999;25(3):123–134.

[15] Gannier A. Summer distribution and relative abundance of delphinids in the Mediterranean Sea. Rev Ecol(Terre Vie). 2005;60:223–238.

[16] Fortuna CM, Canese S, Giusti M, Revelli E, Consoli P, Florio G, et al. An insight into the status of the striped dolphins, *Stenella coeruleoalba*, of the southern Tyrrhenian Sea. J Mar Biol Assoc UK. 2007 Oct;87(05):1321–1326.
